# An estrogen analogue and promising anticancer agent refrains from inducing morphological damage and reactive oxygen species generation in erythrocytes, fibrin and platelets: a pilot study

**DOI:** 10.1186/1475-2867-14-48

**Published:** 2014-06-07

**Authors:** Lisa Repsold, Etheresia Pretorius, Annie Margaretha Joubert

**Affiliations:** 1 Department of Physiology, School of Medicine, Faculty of Health Sciences, University of Pretoria, Pretoria, South Africa

**Keywords:** 2-Methoxyestradiol analogues, 2-Ethyl-3-*O*-sulphamoyl-estra-1, 3, 5(10)16-tetraene, Reactive oxygen species, Cancer

## Abstract

**Background:**

2-Methoxyestradiol is known to have antitumour and antiproliferative action *in vitro* and *in vivo*. However, when 2-methoxyestradiol is orally administered, it is rapidly oxidized by the enzyme 17Î²-hydroxysteriod dehydrogenase in the gastrointestinal tract. Therefore, 2-methoxyestradiol never reaches high enough concentrations in the tissue to be able to exert these antitumour properties. This resulted in the *in silico*-design of 2-methoxyestradiol analogues in collaboration with the Bioinformatics and Computational Biology Unit (UP) and subsequent synthesis by iThemba Pharmaceuticals (Pty) Ltd (Modderfontein, Midrand, South Africa). One such a novelty-designed analogue is 2-ethyl-3-*O*-sulphamoyl-estra-1, 3, 5(10)16-tetraene (ESE-16).

**Methods:**

This pilot study aimed to determine the morphological effect and possible generation of reactive oxygen species by ESE-16 on erythrocytes and platelet samples (with and without added thrombin) by means of scanning electron microscopy, transmission electron microscopy and flow cytometry.

**Results:**

Erythrocytes and platelets were exposed to ESE-16 at a concentration of 180nM for 24Â hours. Scanning- and transmission electron microscopy indicated that ESE-16 did not cause changes to erythrocytes, platelets or fibrin networks. Flow cytometry measurements of hydrogen peroxide and superoxide indicated that ESE-16 does not cause an increase in the generation of reactive oxygen species in these blood samples.

**Conclusion:**

Further *in vivo* research is warranted to determine whether this novel *in silico*-designed analogue may impact on development of future chemotherapeutic agents and whether it could be considered as an antitumour agent.

## Introduction

Cancer is currently one of the leading causes of mortality across the worldâ€™s population [1]. Research aimed at finding possible compounds to treat or cure cancer is therefore of extreme importance. 2-Methoxyestradiol (2ME) (FigureÂ 1) is a metabolite of 17Î²-estradiol which has been tested for its antitumour and antiangiogenic properties *in vitro* and *in vivo*[2,3].

**Figure 1:**
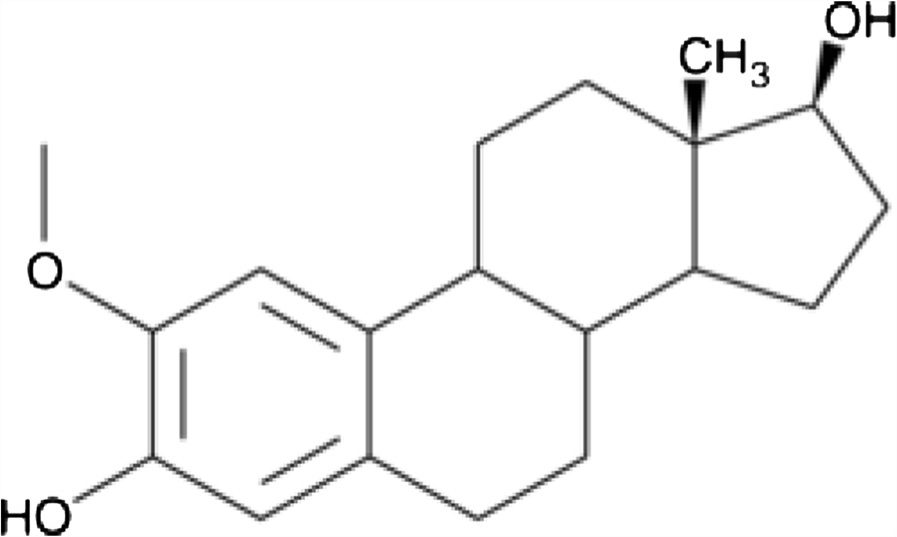
**Structure of 2-methoxyestradiol **[4]**.**

2ME however, has poor bioavailability limiting the action *in vivo* as indicated in earlier phase I studies where 2ME was orally administered to cancer patients in the form of a capsule [5]. The rate at which 2ME becomes available to the site of drug action is low since the rate of oxidation of 2ME is higher than absorption [5]. 2ME is rapidly oxidized in the gastrointestinal tract when orally administered by the enzyme 17Î²-hydroxysteriod dehydrogenase [6,7]. This leads to decreased levels of 2ME, thus being too low to exert significant antitumour effects [8]. The development of a 2ME NanoCrystalÂ® colloidal dispersion (NCD) was subsequently formulated. The latter increased bioavailability, however, the antitumour effects were not optimal in patients [9]. This led to the development and synthesis of analogues of 2ME to test for a compound with increased antitumour and antiangiogenic properties with increased bioavailability [10,11]. Such analogues of 2ME have been *in silico*-designed in our laboratory (FigureÂ 2). One novel *in silico*-designed analogue is 2-ethyl-3-*O*-sulphamoyl-estra-1, 3, 5(10)16-tetraene (ESE-16) (FigureÂ 2).

**Figure 2:**
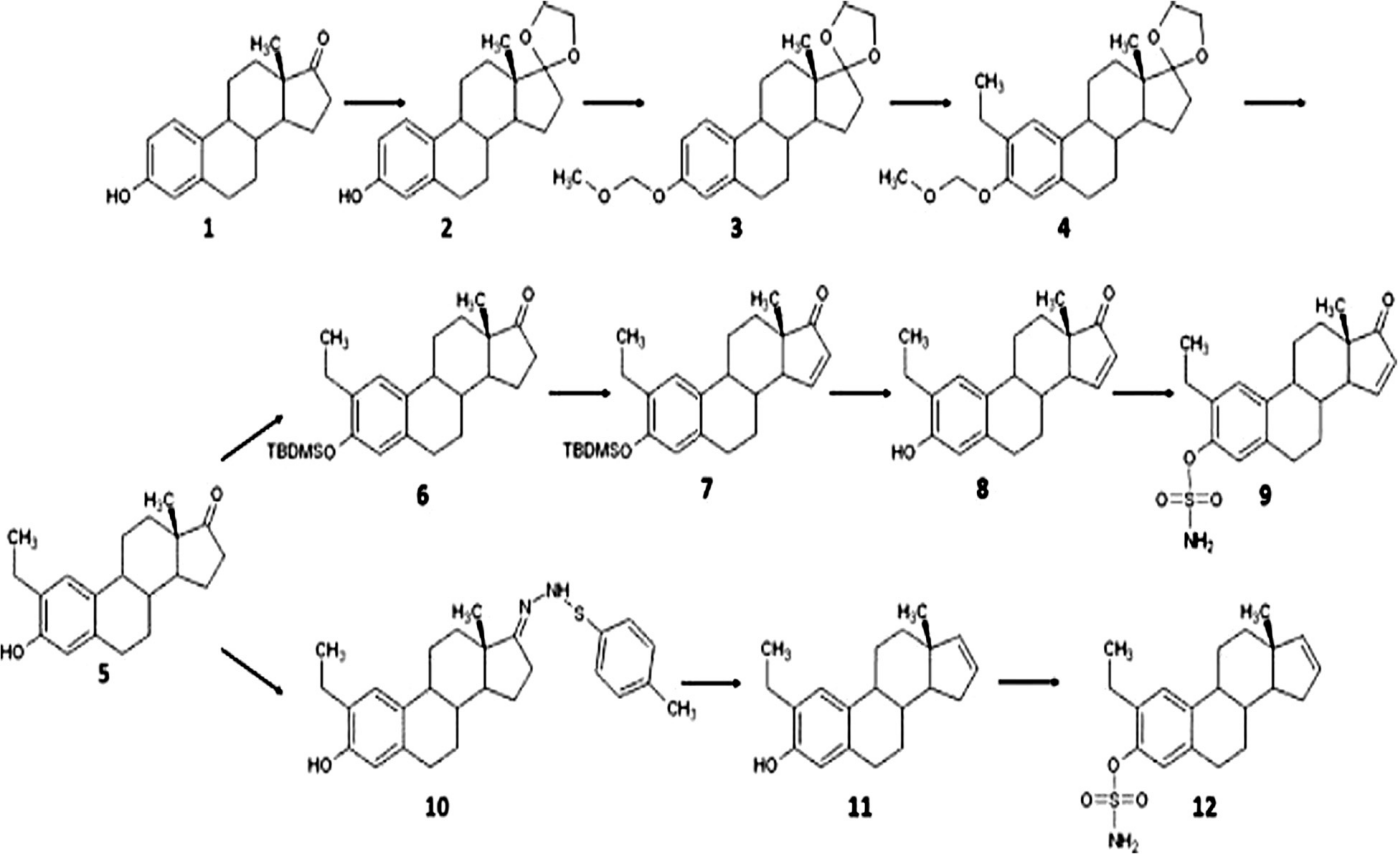
**Synthesis of various sulphamoylated analogues of 2-methoxyestradiol.** Synthesis of 2-ethyl estrone derivatives and the structure of the sulphamoylated compound ESE-16 are indicated in the image in step 12 [12].

2ME exerts its anticancer signaling by disrupting mitochondrial function, generating reactive oxygen species (ROS) and by targeting microtubule dynamics *in vitro* and *in vivo*[13]. 2ME results in apoptosis involving both the extrinsic and intrinsic pathways [14]. 2ME activates apoptosis via the extrinsic pathway by means of death receptors (DRs) such as death receptor 5 (DR5) and activates caspases known to promote the extrinsic pathway [14]. The extrinsic pathway of apoptosis comprises of the instigation of caspase 8, caspase 3, and upregulation of DR5 with activation of caspase 9 present in the intrinsic pathway through crosstalk with caspase 8 [14,15].

Production of ROS in 2ME- and analogue-treated cells is likely to be a result of the inhibition of the mitochondrial electron transport complex I [13] leading to autophagy by means of beclin-1 upregulation and autophagy protein 4 (Atg4) inactivation [16]. Generation of ROS upregulates beclin-1 which results in the increased incidence of autophagy, while hydrogen peroxide causes autophagy and the inactivation of Atg4 responsible for the induction of autophagy [17]. 2ME also induces apoptosis in cancer cells as a result of the upregulation of p53, since p53 is known to arbitrate cell cycle arrest that may lead to the induction of apoptosis by controlling the G_2_/M cell cycle checkpoint [18,19].

Previous morphological studies of 2ME-treated cancer cells have shown the presence of characteristics of apoptosis including cell membrane blebbing, apoptotic bodies, cell shrinkage and hypercondensation of chromatin [20]. The presence of altered chromosomes and spindles has also been morphologically determined and indicates the occurrence of micronuclei, atypical distribution of chromosomes and the formation of multipolar spindles [18,21].

The mechanism of action of 2ME is via impairment of microtubule dynamics by binding to the Î²-tubulinâ€™s colchicine binding site and thereby inhibiting mitotic spindle function, leading to blocking of cells in mitosis and eventually cell death [22-25]. Of the various analogues of 2ME, 2-ethyl derivatives *in silico*-designed, were determined to have increased binding to the colchicine binding site, thus leading to increased disruption of microtubule dynamics in the cell and G_2_/M arrest [26-28].

Previously in our laboratory, it was determined that the newly designed analogues are more potent when compared to 2ME, disrupting Î±-tubulin dynamics resulting in apoptosis and autophagy. The effect of ESE-16 was previously determined on a non-tumorigenic MCF-12A cell line in our laboratory and it was demonstrated that ESE-16 had decreased discernment towards the non-tumorigenic cell line when compared to a cancer cell line [29]. These sulphamoylated analogues have increased anticancer activity as they bind to carbonic anhydrase II (CAII) present in red blood cells delaying early metabolism [12,29].

This preliminary study aimed to determine the influence of ESE-16 on erythrocytes and platelets in terms of morphology and generation of reactive oxygen species (ROS) by means of scanning electron microscopy, transmission electron microscopy and flow cytometry. The time and concentration at which erythrocytes and platelets were exposed were previously established in our laboratory by means of growth inhibition studies (GI_50_) [12]. The effect of ESE-16 on the structure of erythrocytes, platelets and fibrin networks has not yet been determined and as this compound is theorized to be carried in the blood bound to CAII, the effect of ESE-16 on the morphology of erythrocytes, platelets and fibrin networks is significant [12]. This study reveals the *ex vivo* potential of this compound as a study technique to establish the specific mechanisms of action and future studies will determine the involvement of all related systems. Data from this study merits further *in vivo* research to determine the impact of this novel *in silico*-designed analogue on development of future chemotherapeutic agents and whether it could be considered as an antitumour agent.

## Results

### Morphology

#### Scanning electron microscopy

Images obtained from SEM analysis indicated the normal morphology of fibrin networks, platelets and erythrocytes. ESE-16-treated samples showed no damage as seen in positive control samples or deviation from the normal morphology when compared to the control samples (FigureÂ 3).

**Figure 3:**
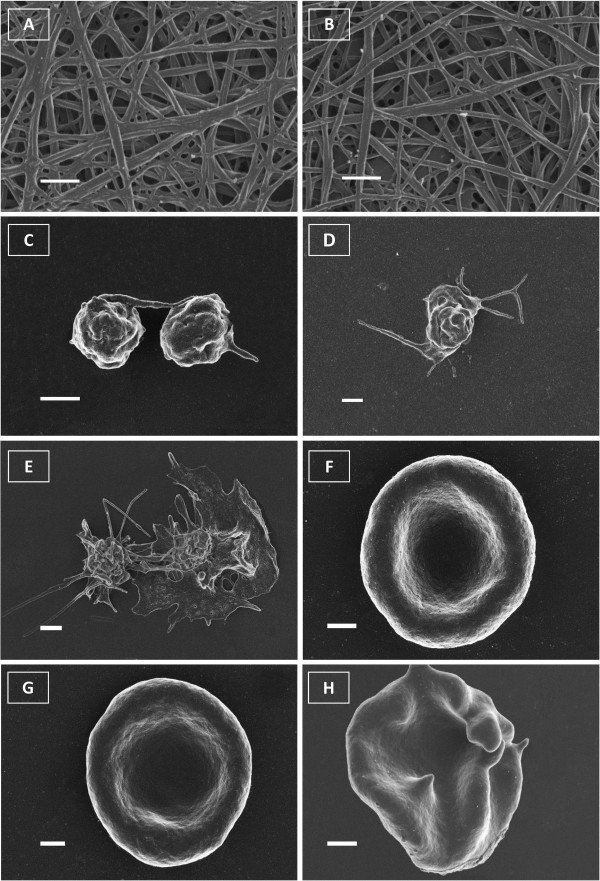
**Scanning electron microscopy images of control and ESE-16-treated fibrin networks, platelets and erythrocytes.** The addition of thrombin to PRP resulted in the formation of fibrin networks (**A** - scale indicates 1 μm). Fibrin networks formed from ESE-16-treated PRP samples indicated normal fibrin morphology (**B** - scale indicates 1 μm). Control plasma smears to show platelet morphology (without thrombin) indicated normal morphology of platelets (**C** - scale indicates 1 μm) which corresponded with the morphology of ESE-16-treated platelets (**D** - scale indicates 2 μm) and is not similar to positive control DMSO-treated platelets which clearly reveals platelet activation (**E** - scale indicates 2 μm). Whole blood samples (prepared from whole blood smears without thrombin) indicated biconcave morphology of control erythrocytes (**F** - scale indicates 1 μm) which resembled the morphology seen in ESE-16-treated erythrocytes (**G** - scale indicates 1 μm) but not that of DMSO-treated erythrocytes at a concentration of 1% as positive control showing signs of oxidative stress with membrane lengthening (**H** - scale indicates 1 μm).

#### Transmission electron microscopy

Illustrations of TEM analysis indicated that ESE-16-treated platelets (FigureÂ 4C and D) were not affected with similar morphology to platelets in control samples (FigureÂ 4A and B) including granularity and membrane integrity. Whole blood control samples (FigureÂ 4A and B) indicated normal morphology of erythrocytes. Whole blood samples exposed to ESE-16 (FigureÂ 4C and D) revealed normal erythrocyte morphology with no sign of distress.

**Figure 4:**
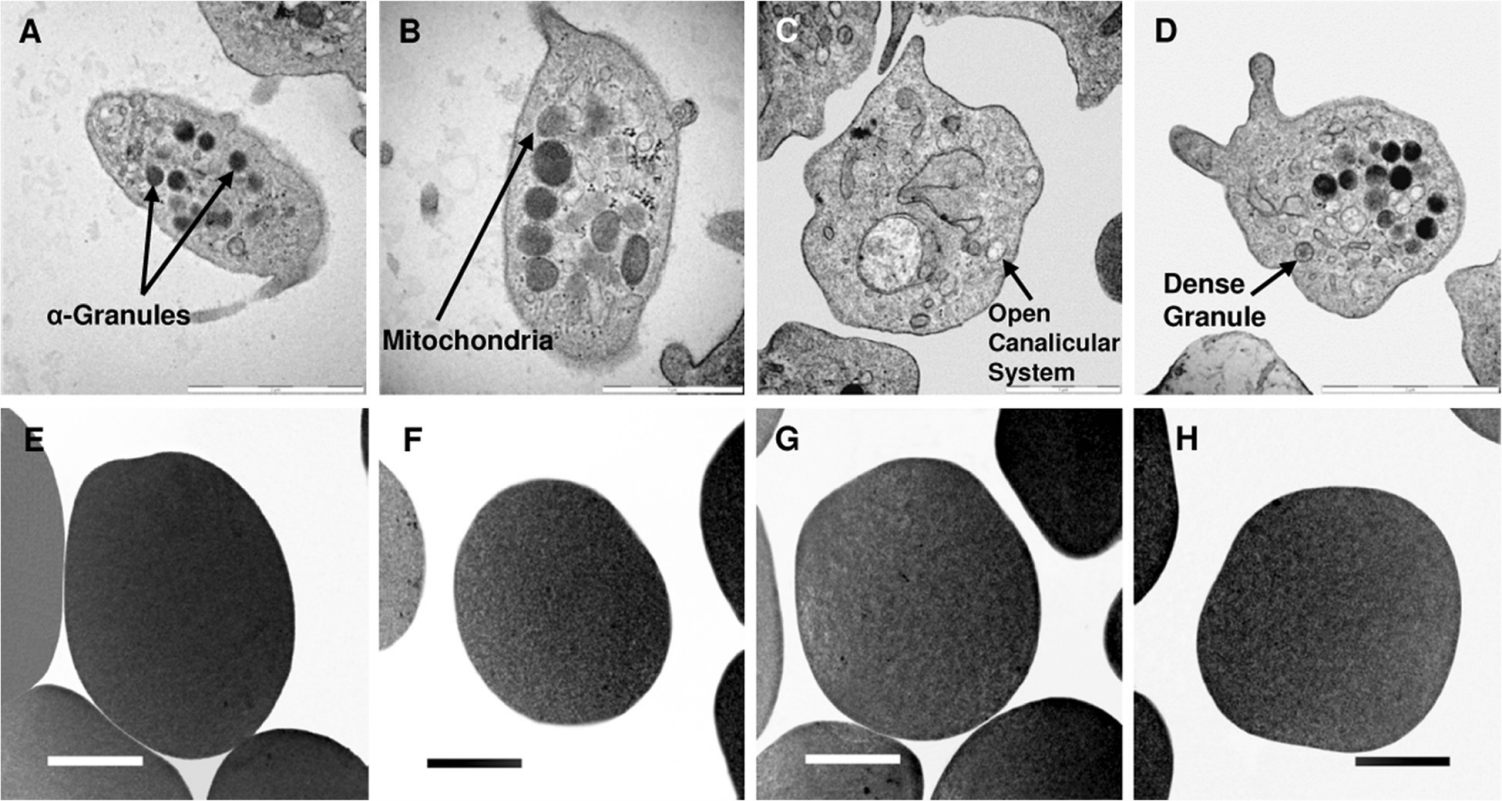
**Transmission electron microscopy images of control and ESE-16-treated platelets and erythrocytes.** Plasma control samples showed normal morphology of platelets including granularity (α- and dense granules) and presence of mitochondria (**A** scale indicates 2 μm &**B** scale indicates 1 μm). Platelets exposed to ESE-16 indicated no effect on platelet morphology or interior cellular structures and granularity of the platelets remained intact (**C** scale indicates 2 μm &**D** scale indicates 1 μm). Whole blood control samples indicated normal morphology of erythrocytes (**E & F** - scale indicates 2 μm). Whole blood exposed to ESE-16 showed no significant damage or structural changes to the morphology or membrane of erythrocytes (**G & H** - scale indicates 2 μm).

### Reactive oxygen species generation

#### Hydrogen peroxide measurement

To examine the generation of hydrogen peroxide after exposure of blood samples to ESE-16 when compared to control- and vehicle-treated samples, flow cytometry was used in combination with the DCFDA probe. Hydrogen peroxide generation was not increased when blood samples were treated with ESE-16 compared to the control samples (FigureÂ 5A) and was validated with the use of Cyflogic version 1.2.1 software (Pertu Therho, Turko, Finland).

**Figure 5:**
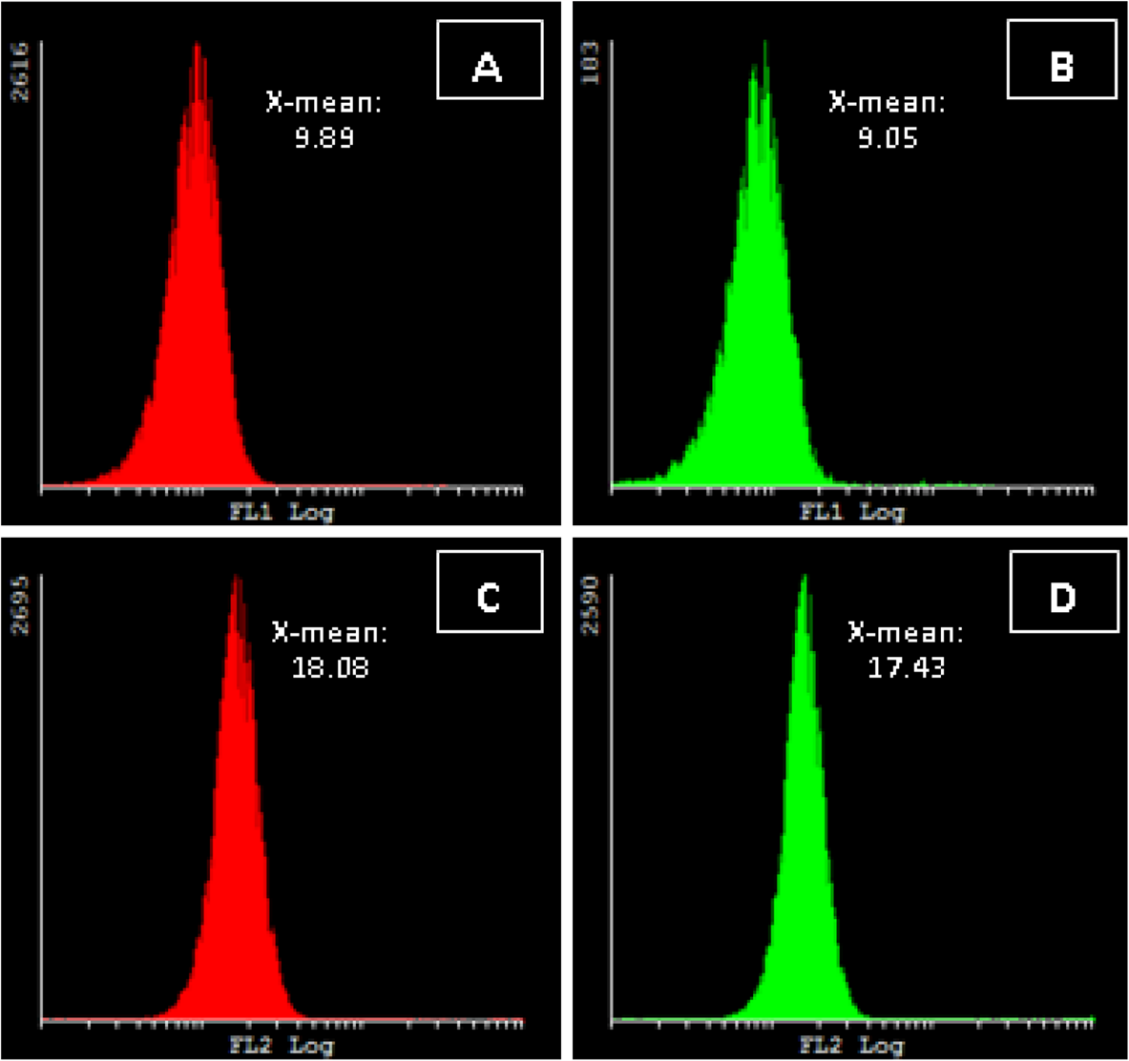
**Measurement of ROS production in control and ESE-16-treated blood samples.** Hydrogen peroxide detection utilizing DCFDA probe of control blood samples **(A)** and ESE-16-treated samples **(B)** showed no statistical difference. Superoxide detection utilizing HE probe indicated control blood samples **(C)** and ESE-16-treated blood samples **(D)** which once again indicated not statistically significant (a *P*-value of less than 0.05 was accepted as statistically significant).

#### Superoxide measurement

Superoxide generated from ESE-16-treated blood samples was determined by means of flow cytometry using the HE probe. Superoxide generation was revealed not to be statistically significantly increased in ESE-16 treated cells (indicated in green graphs) when compared to control samples (indicated in red graphs) (Cyflogic version 1.2.1 software (Pertu Therho, Turko, Finland) (FigureÂ 5B).

## Discussion

The GI_50_ concentration and exposure time of 2-ethyl-3-*O*-sulphamoyl-estra-1, 3, 5(10)16-tetraene (ESE-16) have previously been established in our laboratory [12]. The current study is the first to report on the *ex vivo* effects potentiated by ESE-16. Previous studies suggest that ESE-16â€²s mechanism of action induce both apoptosis and autophagy in the tumorigenic cell lines attributed to the formation of reactive oxygen species.

Apoptosis can be induced by ROS production generated from Fas ligand which binds to Fas receptors and recruits Fas-associating protein with death domain. Caspase 8 culminates in apoptosis and characteristically DNA fragmentation as one of the hallmarks of this type of cell death [30]. ROS generation may also induce autophagy by means of Beclin-1 upregulation, inactivation of autophagy protein 4 (Atg4) and increased expression of autophagy protein 5 (Atg5) which result in self-catabolism of cellular organelles and degradation in lysosomes [30]. The latter accounts for the increased formation of acidic vacuoles characterized by autophagy [16,17,30]. Genes involved in the activation of apoptosis and autophagy caused when MCF-7 cells are treated with ESE-16 was determined with the use of gene and protein microarrays in our laboratory [29]. These studies also revealed that MCF-7-treated cells undergo apoptosis through activation of the c-Jun NH(2)-terminal kinase (JNK) pathway induced by ruptured lysosomes, increased labile iron, ROS production and Bcl-2 phosphorylation [29].

Further data from *in vitro* studies in our laboratory showed that ESE-16 induces apoptosis and generation of ROS in cancer cell lines [12,29,31-33]. ESE-16 has also been tested on the non-tumorigenic MCF-12A breast cells and have shown a higher affinity for the cancerous cell lines when compared to this non-cancerous cell line [31-33]. The current study shows that no morphological characteristics of ROS generation were seen via SEM and TEM or even quantitatively by means of flow cytometry.

Since ESE-16 was designed to ultimately serve as a treatment for cancer, it was imperative to test the effect ESE-16 might have on the morphology of platelets, fibrin networks and erythrocytes when administered and distributed through the circulatory system. The *ex vivo* effects of ESE-16, however, showed no distress or damage to platelets or fibrin networks implying that ESE-16 has no effect on the activation of the coagulation cascade. TEM analysis did not show any deviations from the normal morphology of the erythrocytes. The formation of reactive oxygen species in blood samples when exposed to ESE-16 was not increased and treatment with ESE-16 does thus not result in the generation of ROS in blood samples. From the results it has been determined that ESE-16 does not cause distress when administered *ex vivo* to platelets, erythrocytes or fibrin networks thus indicating the viability of ESE-16 to be applied to *in vivo* studies.

## Conclusion

In conclusion, ESE-16â€²s effects *in vitro* are favorable as a promising antitumour agent causing metaphase block, apoptosis and autophagy in tumorigenic cell lines and not to the same extent in MCF-12A cells. In the current study it was shown that no damage was caused to the morphological structure of platelets, fibrin networks or erythrocytes. Reactive oxygen species was not increased when blood samples were exposed to ESE-16. Data demonstrate the possibility of ESE-16 as a potential anticancer candidate, causing no damage to circulatory components. In the light of these preliminary data future *ex vivo* and *in vivo* research is warranted to investigate whether this novel *in silico*-designed analogue might be considered as an antitumour agent.

### Materials and methods

#### Blood

Blood for *ex vivo* analysis was obtained from 3 healthy males who did not smoke or have not used any medication. Blood was collected in citrate tubes which were acquired from Transpharm (Gauteng, SA). Platelet-rich plasma (PRP) was obtained by centrifuging the blood sample at 1000Â rpm for 2Â min and collecting plasma from the separated blood [34]. Whole blood (200 μl) and PRP (200 μl) were exposed to ESE-16 for 24Â hours in 96-well plates [34]. To prepare fibrin clot or coagulum, human thrombin (20 U/ml) containing 0.2% human serum albumin (obtained from the South African National Blood Service, Gauteng, South Africa) was added to PRP samples and ESE-16-exposed PRP samples to determine whether ESE-16 would have an effect on the coagulation process of plasma [34]. Platelets were studied by making PRP smears on glass cover slips, while erythrocyte morphology were studied by making whole blood smears on glass cover slips (here no thrombin was added).

#### 2-Ethyl-3-*O*-sulphamoyl-estra-1, 3, 5(10)16-tetraene (ESE-16)

ESE-16 is an *in silico*-designed compound derived from 2ME in collaboration with the Bioinformatics and Computational Biology Unit (UP) and is therefore not commercially available. Synthesis was conducted by iThemba Pharmaceuticals (Pty) Ltd (Modderfontein, Midrand, South Africa). The concentration and time at which samples were exposed to ESE-16 was previously determined in our laboratory via growth inhibition 50 (GI_50_) studies and was found to be 180â€‰Â±â€‰10.76 nM [12]. *Ex vivo* blood samples were therefore exposed to 180 nM of ESE-16 for 24Â hours for SEM, TEM and ROS measurement [12].

#### Scanning electron microscopy

*Ex vivo* samples were prepared on glass plates as follows: 10 μl whole blood as a control, 10 μl whole blood with 5 μl thrombin, 10 μl whole blood exposed to ESE-16, 10 μl whole blood exposed to ESE-16 with 5 μl thrombin, 10 μl whole blood exposed to DMSO at a concentration of 1% (v/v) as a positive control. PRP samples composed of 10 μl PRP as a control, 10 μl PRP with 5 μl thrombin, 10 μl PRP exposed to ESE-16, 10 μl PRP exposed to ESE-16 with 5 μl thrombin, and 10 μl PRP exposed to DMSO at a concentration of 1% (v/v) as a positive control. Glass plates with *ex vivo* samples were placed in 6-well plates and left to dry until a film formed, after which the samples were washed for 20Â min in a 50% phosphate buffered saline (PBS): 50% distilled H_2_O solution. Samples were fixed with gluteraldehyde and PBS for 30Â min and washed 3 times in PBS for 3Â min each for subsequent secondary fixation in osmium tetraoxide for 15Â min. Samples were washed 3 times each for 3Â min and dehydrated for 3Â min each in increasing concentration of ethanol, 30%, 50%, 70%, 90% and three times in 100% ethanol [34,35]. Samples were critically dried, mounted and carbon coated and was viewed with the Zeiss ULTRA plus FEG-SEM (Carl Zeiss (Pty) Ltd, Johannesburg, South Africa).

#### Transmission electron microscopy

Whole blood and PRP were obtained and samples were exposed to ESE-16 with appropriate controls included as previously defined. Blood samples were fixed for 30Â min in the same solution and rinsed in 50% PBS: 50% distilled H_2_O solution (three times for 3μ5Â min each). Blood samples were fixed with osmium tetraoxide solution for 15Â min and washed three times with PBS: distilled H_2_O solution (1:1) for 3Â min. Samples were dehydrated by emerging in 30%, 50%, 70%, 90% and three times in 100% ethanol, whereafter samples were embedded in Quetol resin for 36Â hours. Samples were cut with an ultra microtome into ultra-thin sections with a diamond knife and contrasted for 15Â min with 4% uranyl acetate and lead citrate for 10Â min [36]. The JOEL Transmission electron microscope (JEM 2100Â F) situated at the Microscopy and Microanalysis Unit of the University of Pretoria, Pretoria, South Africa was employed to view samples.

### Flow cytometry

#### Hydrogen peroxide measurement

Generation of hydrogen peroxide (H_2_O_2_) produced by whole blood and PRP was assessed using 2,7-dichlorofluorescein diacetate (DCFDA). When DCFDA is oxidized by ROS and peroxides in the samples it is converted to a highly fluorescent derivative of the non-fluorescent DCFDA, namely 2,7-dichlorofluorescein (DCF) [17,37]. After samples including previously-mentioned controls were prepared and exposed as described, 10 μl whole blood and 10 μl PRP in 1Â ml PBS was used with 0.75 μl DCFDA (10Â mM) probe for 20Â min at 37Â°C. Measurement of DCF (FL1) fluorescence was conducted for at least 10 000 events (n valueâ€‰=â€‰3) with a FC500 System Flow Cytometer (Beckman Coulter South Africa(Pty) Ltd.) which is equipped with an 488Â nm excited air-cooled argon laser. Data analysis was conducted with the use of Cyflogic version 1.2.1 software (Pertu Therho, Turko, Finland) [8].

#### Superoxide measurement

Generation of superoxide was measured in whole blood and PRP samples exposed to ESE-16 along with appropriate controls with the use of hydroethidine (HE). HE is only oxidized by superoxide to a red fluorescent compound and not by any other form of ROS including hydrogen peroxide, singlet oxygen, nitrogen radicals or hydroxyl radicals [17,37]. After whole blood and PRP were exposed, 10 μl whole blood and 10 μl PRP were resuspended in 1Â ml PBS and incubated with 0.75 μl HE probe (20Â mM) for 20Â min at 37Â°C. Red fluorescence produced by samples was measured as the HE fluorescent product (FL2) for at least 10 000 events (n valueâ€‰=â€‰3) with a FC500 System Flow Cytometer (Beckman Coulter South Africa(Pty) Ltd.) which is equipped with an 488Â nm excited air-cooled argon laser. Data analysis was obtained with the use of Cyflogic version 1.2.1 software (Pertu Therho, Turko, Finland) [8].

### Statistics

Number of subjects included for this pilot study was corroborated by the Department of Statistics at the University of Pretoria. In addition, and separate from the number of subjects included, quantitative and qualitative techniques were conducted in triplicate. Qualitative data capturing included SEM and TEM images and were confirmed by quantitative data. Quantitative data included measurement of ROS by HE- and DCF- derived fluorescence which was expressed as a ratio of the value measured for the ESE-16-treated cells compared to the vehicle-treated exposed cells defined as mean relative fluorescence. This involved flow cytometry analysis of at least 10 000 events that was repeated thrice and statistical significance was determined by employing Cyflogic version 1.2.1 software (Pertu Therho, Turko, Finland).

### Ethics

Ethical clearance for the collection of blood was obtained from The Research Ethics Committee, Faculty Health Sciences, University of Pretoria which complies with ICH-GCP guidelines (Ethics clearance number: 151/2006, re-approved 2009).

## Competing interests

The authors declare that they have no competing interests.

## Authors’ contributions

LR was responsible for experimental design, carried out all experiments (SEM, TEM and flow cytometry) and drafting of the manuscript. RP and AMJ was responsible for conceiving the study, participating in the studies design and coordination as well as drafting of the manuscript. All authors read and approved the final manuscript.
